# Transition of Renal Patients Using AlloSure Into Community Kidney Care (TRACK): Protocol for Long-Term Allograft Surveillance in Renal Transplant Recipients

**DOI:** 10.2196/25941

**Published:** 2021-03-15

**Authors:** Bethany L Dale, Subhasish Bose, Sheng Kuo, Alana Burns, Pierre Daou, Jenna Short, Jake Miles

**Affiliations:** 1 CareDx Brisbane, CA United States; 2 Lynchburg Nephrology Physicians Charlottesville, VA United States; 3 Division of Nephrology Department of Medicine University of Virginia Medical Center Charlottesville, VA United States; 4 Nephrology Associates PC Queens, NY United States

**Keywords:** donor-derived cell free DNA (dd-cfDNA), molecular inflammation, molecular injury, acute rejection, allograft injury, allograft surveillance, renal transplant, renal, transplant, injury, graft rejection, kidney, kidney disease, transplantation

## Abstract

**Background:**

Patients with end-stage kidney disease require complex and expensive medical management. Kidney transplantation remains the treatment of choice for end-stage kidney disease and is considered superior to all other modalities of renal replacement therapy or dialysis. However, access to kidney transplant is limited by critical supply and demand, making it extremely important to ensure longevity of transplanted kidneys. This is prevented through lifelong immunosuppression, with caution not to overly suppress the immune system, resulting in toxicity and harm. Transition of care to community nephrologists after initial kidney transplantation and monitoring at a transplant center is an important process to ensure delivery of effective and patient-centric care closer to home. Once transplanted, laborious surveillance of the immune system and monitoring for potential rejection and injury are undertaken through an armamentarium of screening modalities. Posttransplant surveillance for kidney function and injury remains key to follow-up care. While kidney function, quantified by estimated glomerular filtration rate and serum creatinine, and kidney injury, measured by proteinuria and hematuria, are standard biomarkers used to monitor injury and rejection posttransplant, they have recently been demonstrated to be inferior in performance to that of AlloSure (CareDx Inc, Brisbane, CA) circulating donor-derived, cell-free DNA (dd-cfDNA).

**Objective:**

The outcomes and methods of monitoring renal transplant recipients posttransplant have remained stagnant over the past 15 years. The aim of this study is to consider intensive surveillance using AlloSure dd-cfDNA in an actively managed protocol, assessing whether it increases long-term allograft survival in kidney transplant recipients compared with current standard clinical care in community nephrology.

**Methods:**

The study protocol will acquire data from a phase IV observational trial to assess a cohort of renal transplant patients managed using AlloSure dd-cfDNA and patient care managers versus 1000 propensity-matched historic controls using United Network for Organ Sharing U.S. Scientific Registry of Transplant Recipients data. Data will be managed in a centralized electronic data server. The primary outcome will be superior allograft survival, as a composite of return to dialysis, retransplant, death due to allograft failure, and death with a functional graft (infection, malignancy, and cardiovascular death). The secondary endpoints will assess improved kidney function through decline in estimated glomerular filtration rate and immune activity through development of donor-specific antibodies.

**Results:**

The total sample is anticipated to be 3500 (2500 patients managed with AlloSure dd-cfDNA and 1000 propensity-matched controls). Active enrollment began in November 2020.

**Conclusions:**

Based on a significant literature base, we believe implementing the surveillance of dd-cfDNA in the kidney transplant population will have a positive impact on graft survival. Through early identification of rejection and facilitating timely intervention, prolongation of allograft survival versus those not managed by dd-cfDNA surveillance protocol should be superior.

**International Registered Report Identifier (IRRID):**

PRR1-10.2196/25941

## Introduction

The recent presidential executive order to advance American kidney health has directed the U.S. Department of Health and Human Services to increase the number of patients with new end-stage kidney disease (ESKD) to either receive dialysis at home or receive a transplant, with the aim to double the number of kidneys transplanted by 2030 [1].

With over 700,000 patients living with ESKD in the United States, ensuring allograft longevity post-kidney transplant is critical [2]. As compared with dialysis, kidney transplantation is known to provide superior quality of life, patient survival, and cost-effectiveness [3-6].

Minimally invasive biomarkers to detect allograft injury and rejection have become increasingly important as clinicians strive to develop new strategies for personalization of medicine and prolonging allograft survival. Since its discovery in 1948, cell-free DNA has had a significant impact on molecular diagnostics and is now utilized frequently in prenatal genetic screening, preclinical neoplasia detection, and more recently, solid organ transplantation [7]. In recent years, donor-derived, cell-free DNA (dd-cfDNA) has shown clinical validity as a leading biomarker of allograft inflammation and injury [8-11].

In the Circulating Donor-Derived Cell-Free DNA in blood for diagnosing Acute Rejections in Kidney Transplant Recipients (DART) study, Bloom et al [8] assessed 102 paired renal allograft biopsies with dd-cfDNA. The median levels of dd-cfDNA in patients with a histological diagnosis of active rejection were significantly elevated compared to those without active rejection (1.6% versus 0.3%, *P*<.01). The receiver-operating characteristic area under the curve was reported at 0.74, which significantly outperformed serum creatinine at 0.54 in detecting active rejection. The performance characteristics of this assay were improved when discriminating antibody-mediated rejection (ABMR) from no ABMR, with an area under the curve of 0.87 [8]. Huang et al [10] validated these findings in a single-center study assessing 63 highly sensitized renal transplant patients with paired allograft biopsies and AlloSure dd-cfDNA.

More recently, in the Resolution by AlloSure Differentiates Ambiguous Rejection (RADAR) study, Stites et al found that AlloSure dd-cfDNA >0.5% can aid in the risk stratification of patients with T-cell mediated rejection grade 1A or borderline rejection with respect to poor clinical outcomes identified as estimated glomerular filtration rate (eGFR) decline, de novo donor-specific antibody (DSA) formation, and recurrent rejection episodes [11].

AlloSure dd-cfDNA has also shown associations with de novo DSA formation and ABMR previous to RADAR. Jordan et al [12] identified 90 dd-cfDNA samples paired with DSAs and clinically indicated biopsies and demonstrated the combination of dd-cfDNA and DSA testing improved the diagnostic yield of noninvasive diagnosis of ABMR to 89% positive predictive value.

Understanding the severity of opportunistic infections in immune-suppressed patients resulting in kidney injury is another area of utility. dd-cfDNA has been demonstrated to aid in differentiation of BK viremia and BK nephropathy, as well as help in cases with high viral copy number and confounding biopsy results [13,14].

Further, it is estimated that 60% of late rejections and 30% of early rejections can be attributed to posttransplant nonadherence to immunosuppressants. A meta-analysis and systemic review demonstrated patients who receive adherence intervention have significantly higher compliance (odds ratio=2.366) [15]. Transplant centers currently utilizing patient care managers (PCMs) also have improvements in patient adherence, upwards of 30% (unpublished data). These findings, in conjunction with other supporting evidence, demonstrate that the analysis of dd-cfDNA and PCM support are helpful in the assessment of transplant patients and provide additional information of allograft status.

In addition to allograft rejection, the clinical significance of other morbidity-affecting outcomes remains significant. Malignancy, infection, cardiovascular complications, and bone complications are an inevitable consequence of a lifetime of immunosuppression, adding a significant burden on patients and the health care system. Management of these patients is complex and often multidisciplinary, where AlloSure can provide additional, previously lacking, information about the allograft. This was recently demonstrated in a case report by Lipson et al [16] in the management of a renal transplant recipient (RTR) on checkpoint inhibitors to treat malignancy.

Death with a functioning graft (DWFG) accounts for 47% of all transplant losses after 10 years. Additionally, registry data and retrospective analyses of long-term outcomes from randomized trials have highlighted cardiovascular disease, followed by malignancy as the top causes of morbidity and DWFG. However, while death from cardiovascular disease in RTRs appears to be declining, mortality from malignancy is increasing [17].

The increasing number of transplants worldwide is further resulting in a growing cohort of posttransplant recipients (179,361 RTRs in the United States in 2010); due to the limited number of transplant nephrologists, these patients are increasingly likely to encounter practitioners in other specialties. Thus, understanding the value of AlloSure dd-cfDNA in the context of long-term management is an area of key importance. The aim of this study is to assess the value of AlloSure dd-cfDNA and an actively managed protocol in the context of chronic complications and determine how it may influence long-term outcomes relevant to generalists.

As the number of kidney transplants performed continues to increase in the United States, so does the cohort of long-term RTRs, which was estimated at over 220,000 in 2017 [18]. With limited transplant nephrologists and hospital outpatient resources, many of these patients will be managed under a shared care model between community physicians and transplant centers. The Transition of Renal patients using AlloSure into Community Kidney care (TRACK) study aims to assess the utility of AlloSure dd-cfDNA surveillance and supplemental patient care management for RTRs in prolonging allograft survival and improving long-term clinical outcomes including graft function and immunological status.

## Methods

### Study Design

This is a phase IV, prospective, multicenter, observational, cohort study designed to evaluate the effectiveness of AlloSure dd-cfDNA (CareDx Inc, Brisbane, CA) surveillance and patient care management in kidney transplant patients in prolonging allograft survival, allograft function, and immunological status. All prospective data will be collected from patient electronic medical records. Propensity-matched control data will be sourced from United Network for Organ Sharing U.S. Scientific Registry of Transplant Recipients databases. The study duration will be event driven, with each patient’s enrollment lasting 5 years.

### Outcomes and Measures

The primary endpoint of this study will be superior graft survival measured as the time to allograft loss, defined as the composite of return to dialysis, retransplant, death due to allograft failure, and DWFG (infection, malignancy, and cardiovascular death).

Secondary endpoints include assessment of allograft function and immunological status. Allograft function will be measured as the relative change in eGFR from baseline between the 2 study groups: dd-cfDNA surveillance compared to controls. Immunological status will be defined by detection of de novo DSA formation in patients monitored using dd-cfDNA compared to the matched controls.

All clinical events and investigation results will be captured through the patient electronic medical record or via AlloCare, an optional smartphone-based application that provides a patient-driven platform to manage medications, access results, monitor wellness activities, and streamline communication with their care team. Patients will also be offered mobile phlebotomy when they are unable to visit a local laboratory. The mobile blood draw will be coordinated by CareDx, which will draw all regular tests, urine samples, as well as the AlloSure. Support for testing adherence will be provided by PCMs (CareDx Inc, Brisbane, CA) to assist with scheduling both in-center and mobile draws and provide laboratory visit reminders.

### Testing Schedule

**Figure 1:**
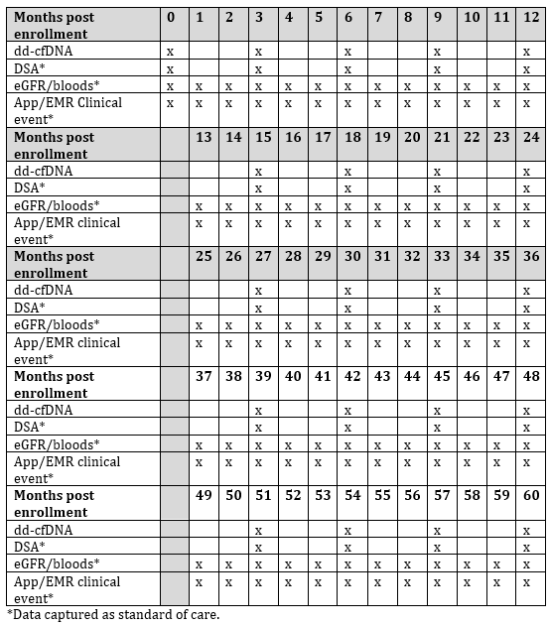
Schedule of testing events. dd-cfDNA: donor-derived cell-free DNA; DSA: donor-specific antibody; eGFR: estimated glomerular filtration rate; EMR: electronic medical record.

Patients will have quarterly AlloSure dd-cfDNA testing (every 3 months) as part of their posttransplant surveillance for a period of 5 years. DSA, eGFR, routine transplant bloods, and clinical events will be captured using the standard of care regime as per institutional guidelines (Figure 1).

In the event of an allograft biopsy (surveillance or for-cause), dd-cfDNA testing is suggested to be drawn prior to the biopsy. For patients with a histological diagnosis of allograft rejection and who are hospitalized, dd-cfDNA are suggested to be measured on alternative days while inpatient and then paired with routine blood tests in the immediate 12 weeks following discharge with the goal to assess the response to treatment.

### Patient Care Managers

PCMs will work directly with participating centers to assist with study protocol adherence and support the integration of the AlloCare phone application. PCMs will coordinate mobile home phlebotomy draws, provide patient education, maintain AlloSure standing order requests in line with the study testing schedule, and provide logistic support to both centers and patients alike.

### Rationale for Testing Schedule

The rationale of the quarterly assessment of dd-cfDNA in the context of renal transplantation is based on key milestones in the posttransplant care of patients and aligns with the routine testing for baseline transplant bloods, urinalysis, and DSA monitoring. Commencing periodic dd-cfDNA surveillance at the 6-month time point posttransplant also complements the transition of care back to community nephrologists for many patients.

Antibody-mediated rejection is widely recognized as the leading cause of transplant failure and accounts for approximately two-thirds of renal allograft losses. Clinical studies over the last 10 years have established that antibodies generated de novo posttransplantation against DSAs are strongly associated and may be an important cause of allograft loss. Detection of a significant level of de novo DSAs should prompt verification of medication compliance and identification of potential sensitization events to minimize the risk of future rejection episodes [19]. Additionally, new data suggest that the elevation of dd-cfDNA precedes de novo DSA formation and correlates with increasing mean fluorescence intensity [12,20]. With the increasing risk of ABMR, assessing dd-cfDNA at these intervals allows practitioners to identify early molecular allograft injury.

Preliminary data are suggestive that elevated dd-cfDNA within the first year posttransplant is associated with significant eGFR decline of 25% in the subsequent year [21]. Quarterly surveillance allows identification of patients that may require increased surveillance and intervention while allowing longitudinal assessment to discern if these trends are appreciated during long-term follow-up.

### Participants

All patients who underwent a kidney transplant within ≥6 months and ≤18 months will be screened to identify patients who are eligible for the study based on inclusion and exclusion criteria. Patients who are eligible to enter the study will be approached for consent during their routine posttransplant clinic visits and will be considered enrolled when they have signed the informed consent form. Selection criteria are shown in Textbox 1.

**Study selection criteria.**

**Inclusion Criteria**

- First draw for purposes of this study ≥6 months and ≤18 months from date of transplant
- Kidney transplant recipient (retransplant and dual kidney permitted)
- Patient’s health care provider adopts and intends to apply the AlloSure Routine Testing Schedule (quarterly draws)
- Participant is willing and able to give informed consent for participation in the trial
- Male or female, aged 12 years or older
- In the investigator’s opinion, patient is able and willing to comply with all trial requirements

**Exclusion Criteria**

- Participant who is pregnant, lactating, or planning pregnancy during the trial
- Significant hepatic impairment (determined by the principal investigator)
- Participant with life expectancy of <6 months or inappropriate for diagnostic monitoring through regular blood sampling
- <6 months and >18 months posttransplant
- Any other significant disease or disorder which, in the opinion of the investigator, may either put the participants at risk because of participation in the trial or may influence the result of the trial or the participant’s ability to participate in the trial
- Participants who have participated in another research trial involving an investigational product in the past 12 weeks
- Recipients of multiorgan transplant (eg, kidney-pancreas)
- Recipients of a transplant from a monozygotic (identical) twin
- Recipients of nonautologous bone marrow transplant
- Patients with a history of poor compliance or needle phobia

A 2-sided log-rank test with an overall sample size of 3500 kidney transplant patients (of which 2500 will be periodically assessed with AlloSure and 1000 are matched controls receiving standard of care) achieves 87% power at a 5% significance level to detect an allograft survival difference of 5% (ie, 75%, AlloSure assessed; 70%, standard of care) during the 5-year surveillance period. This corresponds to a hazard ratio of 0.807, which is a 19.3% reduction in the risk of allograft loss in the AlloSure-assessed patients in comparison to standard of care. There is no consideration of patients lost to follow-up due to the United Network for Organ Sharing U.S. Scientific Registry of Transplant Recipients database, which tracks transplanted organ survival for all organ transplant recipients; however, this analysis does account for an attrition rate of 65%.

A decline in GFR has been shown to be a valid surrogate for long-term outcome in renal transplantation. A 2-sided *t* test or its nonparametric analog will be used to assess differences in the distribution of 5-year change from baseline in eGFR between AlloSure and standard of care surveillance groups. These tests will have approximately 76% power at a 5% significance level to detect a 25% difference in eGFR decline over the 5-year surveillance period. This assumes, over the 5-year surveillance period, an average of 12 mL/min per 1.73 m^2^ decline in eGFR for standard of care and an average 9 mL/min per 1.73 m^2^ decline in eGFR for AlloSure (both groups having similar standard deviations of 30 mL/min per 1.73 m^2^).

Assuming the proportion of control subjects with formation of de novo DSA antibodies during the 5-year surveillance period is 40%, a 2-sided Z-test with continuity correction and pooled variance with the aforementioned sample size of 3500 achieves 78% power at a 5% significance level to detect a de novo DSA formation difference of 5% (ie, 35%, AlloSure assessed; 40%, standard of care) during the 5-year study surveillance period.

### Statistical Analysis

Data will be assessed for normalization and are likely to be nonparametric. Appropriate statistical tests will be applied with the final analysis occurring at the end of the study. Statistical assessments resulting in a *P* value <.05 will be deemed significant. All participants who have at least one AlloSure assessment during the surveillance period will be included in the analysis.

## Results

This study received Western Internal Review Board approval in September 2020. A total of 20 community nephrology practices are expected to participate in this study. Active enrollment began in November 2020. Study insights and conclusions are expected to be presented intermittently throughout the study at international conferences and manuscripts submitted to peer-reviewed academic journals.

## Discussion

Patients undergoing kidney transplantation (either de novo or retransplant) are routinely surveyed with interval blood tests as part of standard postoperative care through outpatient consultation. These tests include serum creatinine, immunosuppressive drug levels, complete blood count, urinalysis, and DSA testing at various intervals. The ability to screen patients to accurately risk-stratify those likely to develop an adverse event using dd-cfDNA is likely to be advantageous, with the potential to improve graft survival and outcomes for transplant patients.

As we evolve our understanding of dd-cfDNA as a leading indicator of poorer outcomes, monitoring longitudinal trends of dd-cfDNA may help risk-stratify the patient population prior to development of graft dysfunction. Elevations in dd-cfDNA are a complementary indicator of graft health and immunological quiescence, helping to further augment and improve our current assessment capabilities and conceivably facilitate earlier intervention. Additionally, the levels of dd-cfDNA can track real-time response to treatment due to a short half-life, be used as an adjuvant marker with histological findings to predict eGFR decline, stratify patients that are likely to develop de novo DSA, and allow optimization of immunosuppression safely over time [11,22,23].

PCM support is designed to facilitate protocol adherence and assist with scheduling mobile blood draws for surveillance testing. Analogous to the function of transplant coordinators, PCMs can support patients with education, appointment scheduling, logistical solutions, and coordination of care to drive adherence and improvements in delivery of posttransplant care. Maintaining protocol adherence aims to enhance allograft surveillance and identify clinical events early, allowing for tailored interventions to improve patient outcomes.

By providing a surveillance tool like AlloSure and patient care management to community nephrologists via TRACK, we can allow a more comprehensive assessment of the patient and allograft beyond the traditional measures such as eGFR, creatinine, and DSA values. With TRACK, when a patient has transitioned to a community center, they will continue to have access to dd-cfDNA testing to monitor allograft status. Longitudinal surveillance of dd-cfDNA will provide an understanding of patient-specific baselines, which serve as a reference point to identify actionable changes in dd-cfDNA that may indicate significant clinical events requiring further investigations and intervention.

This study will also be conducted over a duration of 5 years postenrollment. While the 1-year survival of many kidney transplants is quite good, those further from transplant have worsening survival. This extended timeline is essential to understanding allograft survival, as there is a rapid decline in allograft failure after 3 years posttransplant. Therefore, the longer-term survival of those managed with AlloSure dd-cfDNA and close patient care management will be the primary aim of this study.

While a vast majority of clinical practice adheres to a standard schedule, transplantation is unique in that the timeframe and the testing modality schedule are specific to each transplant center. This heterogeneity is further divided once a patient transfers from the transplant center to the community practice. With this patient population, we acknowledge not every patient will be monitored for DSA or standard labs at the same intervals. The data capture system will be designed to accommodate a quarterly window for the labs that are available to correlate with the AlloSure dd-cfDNA draws.

The TRACK study will assess the validity of dd-cfDNA surveillance and patient care management in kidney transplant patients, aiming to demonstrate superior allograft survival, graft function, and immunological status. This will provide important insights into risk factors for poor clinical outcomes, detect molecular allograft injury and rejection, and identify opportunities for early intervention.

## Abbreviations

ABMR: antibody mediated rejection
DART: Circulating Donor-Derived Cell-Free DNA in blood for diagnosing Acute Rejections in Kidney Transplant Recipients
dd-cfDNA: donor-derived cell-free DNA
DSA: donor-specific antibody
DWFG: death with functioning graft
eGFR: estimated glomerular filtration rate
ESKD: end-stage kidney disease
PCM: patient care manager
RADAR: Resolution by AlloSure Differentiates Ambiguous Rejection
RTR: renal transplant recipient
TRACK: Transition of Renal patients using AlloSure into Community Kidney care
